# 

**Published:** 1849-10

**Authors:** 


DELINQUENCIES.

We are requested by the Publishers and Proprietors of the Journal to call the attention of subscribers to the "beggarly list" of payments to be found on the cover of this number. We cannot doubt that a sight of that list will speak more emphatically to the delinquent than any words that we could use. There it stands.

### *Receipts of Medical Journal for September*, 1849.

Dr. W. A. Ferring, Columbus, Ky. - - - - - - - - $5 00
Dr. Ross, Bolivar, Miss., - - - - - - - - - - - 5 00

The WESTERN JOURNAL OF MEDICINE and SURGERY is published monthly by the undersigned, at the corner of Main and Fifth streets, Louisville, at $5 per annum, payable in advance. Each number contains 96 pages, making two volumes in the year of more than 550 pages each.

Contributions to its pages by the Physicians of the Valley of the Mississippi addressed tothe Editors, are respectfully solicited.

Letters on business, to be addressed, postage paid, to the publishers.

Jauuary, 1844. PRENTICE & WEISSINGER.

